# The effects of dietary proline, β-alanine, and γ-aminobutyric acid (GABA) on the nest construction behavior in the Oriental hornet (*Vespa orientalis*)

**DOI:** 10.1038/s41598-022-11579-w

**Published:** 2022-05-06

**Authors:** Sofia Bouchebti, Levona Bodner, Maya Bergman, Tali Magory Cohen, Eran Levin

**Affiliations:** 1 School of Zoology, Faculty of Life Sciences, Tel Aviv University, 6997801 Tel Aviv, Israel; 2 Steinhardt Museum of Natural History, Tel Aviv University, 69978 Tel Aviv, Israel

**Keywords:** Animal behaviour, Animal physiology, Entomology, Ecology, Zoology

## Abstract

Adult wasps primary food resource is larval saliva. This liquid secretion consists mainly of amino acids and carbohydrates processed from the prey brought to the colony by the foragers. However, adults also regularly consume floral nectar. The nectar's most abundant proteinogenic amino acid is proline, and the two most abundant non-proteinogenic amino acids are β-alanine and GABA. These three amino acids are also common in larval saliva. Here, we study the effect of these dietary amino acids on the physiology and nest construction behavior of the Oriental hornet. Our results reveal their deleterious effects, especially at high concentrations: β-alanine and GABA consumption reduced the hornets' lifespan and completely inhibited their construction behavior; while proline induced a similar but more moderate effect. At low concentrations, these amino acids had no effect on hornet survival but did slow down the nest construction process. Using carbon isotopically labeled amino acids, we show that, unlike proline, β-alanine is stored in most body tissues (brain, muscles, and fat body), suggesting that it is rapidly metabolized after consumption. Our findings demonstrate how a single amino acid can impact the fitness of a nectarivore insect.

## Introduction

The quality of nutrition is often translated to a better survival and reproduction of the individual^1–3^. During the plant-pollinator co-evolution, it has been suggested that plants optimized the composition of their nectar to fit the requirements of nectarivores, in order to achieve effective pollination^4^. Floral nectar contains mainly water and carbohydrates but often also has small quantities of amino acids and secondary compounds such as alkaloids, phenols, and glycosides^5–9^. It has been previously demonstrated that nectarivore insects usually prefer nectar containing amino acids rather than just sugars^10,11^. While the overall composition of the amino acids in the nectar is conserved, their concentrations vary among different plant species^7^. In addition to the 20 amino acids involved in building proteins (proteinogenic amino acids, PAAs), some non-protein amino acids (non-proteinogenic amino acids, NPAAs) can also be found in nectars^5,6^. Whereas PAAs affect the physiology and lifespan of insects^12–14^, little is known about the effects of NPAAs^15^. This study aims to investigate the impact of the three most abundant PAAs and NPAAs found in nectar on the Oriental hornet (*Vespa orientalis*).

Hornets represent an attractive model for the study of nutrition as, unlike other nectarivore insects such as bees or butterflies, plants are not their primary food resource. Hornet workers regularly drink floral nectar and occasionally rob honey from beehives^16^. However, their main food sources are invertebrates and dead vertebrates which they collect to feed their larvae^17^. Hornet larvae can be considered as the super-organism “liver”. They process the protein in the prey and deliver it back to the adults as a drop of saliva containing carbohydrates and free amino acids^18^. The amino acid composition of larval saliva is conserved among hornet species^19^ and is generally richer in concentration and diversity than most nectars^20^.

Oriental hornets construct complex nest structures, including several brood combs, each containing numerous hexagonal cells facing downward^21^. The organic and mineral materials used for the construction are glued together by a cement produced by the adults’ saliva, adding resistance and stability to the combs^22^. Many factors can affect nest construction, such as gravity and the magnetic field^23–25^. Nutrition is also a major influence on nest construction: whereas protein and sucrose ingestion are crucial for construction initiation, mannose and galactose ingestion delay it^21^.

Among PAAs, proline is one of the most abundant amino acids found in the nectar of plants^5,26^. Interestingly, this non-essential PAA is also the most abundant amino acid in the insect hemolymph^12,27^ and in the saliva of several hornet species larvae^19,28^. Proline is also found at high concentrations in honey bees’ honey^29^. It is involved in many physiological processes, including glucogenesis and lipogenesis^30^. It can be rapidly metabolized by the enzyme proline dehydrogenase (POX), providing reducing equivalents and electrons directly to the electron transport system in the mitochondria; and its oxidation enhances carbohydrate metabolism^31^. Proline is therefore commonly used as fuel for flight in different orders of insects^31^. Additionally, proline consumption enhances insect thermotolerance^32,33^ and seems also to be required for egg-laying in honey bee queens^34^. Although honey bees prefer nectar rich in proline^35,36^, the effect of this amino acid on nectarivore insects’ behavior and survival remains unknown.

β-Alanine and γ-aminobutyric acid (GABA) are the two most abundant NPAAs found in plant nectars^5,6^. These two NPAAs are also found in the insect hemolymph^27^, in the larva saliva of hornets^19^, in the honey bees’ honey^29^, and are abundant in the insect nervous system^15^. However, the role of these two NPAAs in insects is still not clear. Since they could potentially affect the behavior of nectarivore insects, their function could be adaptive^15^. GABA is an important inhibitory neurotransmitter in both vertebrates and invertebrates^15^. β-Alanine is the limiting precursor of carnosine synthesis (an intramuscular buffer) and activates GABA receptors^15^. Under natural conditions, nectarivore insects seem to be attracted to nectars rich in GABA and to be repulsed by those rich in β-alanine^11^. However, in a preference test using artificial nectars, foraging honey bees avoided the nectars that contained GABA^36^. The effects of the ingestion of these two NPAAs seem to be species-dependent: GABA increased survival in the bumble bee *Bombus terrestris* and increased locomotor activity in the red mason bee *Osmia bicornis*. Conversely, β-alanine reduced the lifespan in the red mason bee and increased locomotor activity in the bumble bee^37,38^.

Here, we sought to investigate the effects of proline, β-alanine, and GABA, three abundant PAA and NPAAs found in nectar, honey, and hornet larval saliva, on the behavior and physiology of the Oriental hornet. Since the concentrations of amino acids in floral nectar greatly vary from 0 to 1.697 M according to the plant species^6,7^, we tested their effects on hornets at two different concentrations (low (1 mM) and high (1 M) concentrations). First, we fed foragers with a sucrose solution containing one of these three amino acids at the two different concentrations and recorded their survival, nest construction behavior, and food and water consumptions. In the second experiment, we tested the assimilation and allocation of the isotopically labeled amino acids, proline or β-alanine, in different tissues (brain, muscles, fat body, and ovaries) of the hornets.

## Results

### Experiment 1: Hornet nest construction behavior, survival, and nutrient and water consumptions

**Figure 1:**
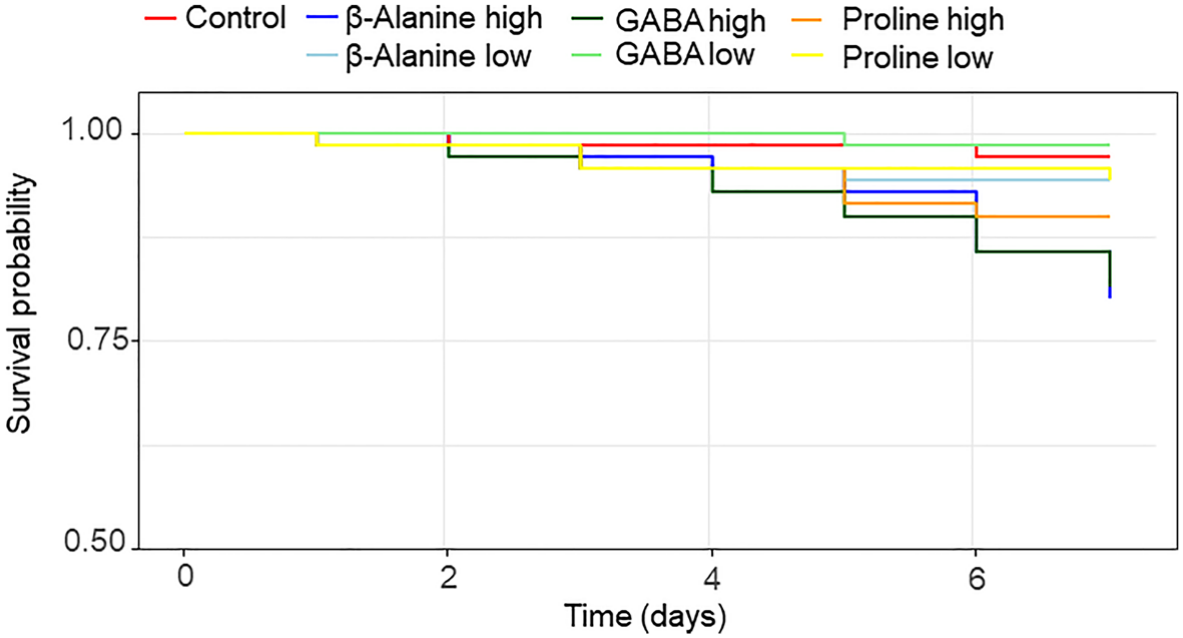
Effect of the amino acids at two different concentrations in the diet (low: 1 mM, and high: 1 M) on the survival.

The concentration of amino acids affected survival (Fig. 1, amino acids × concentration: z = − 1.13, P = 0.260; amino acids: z = 0.32, P = 0.750; concentration: z = 2.67, P < 0.01), with greater mortality seen in hornets fed with a high concentration of amino acids.

**Figure 2:**
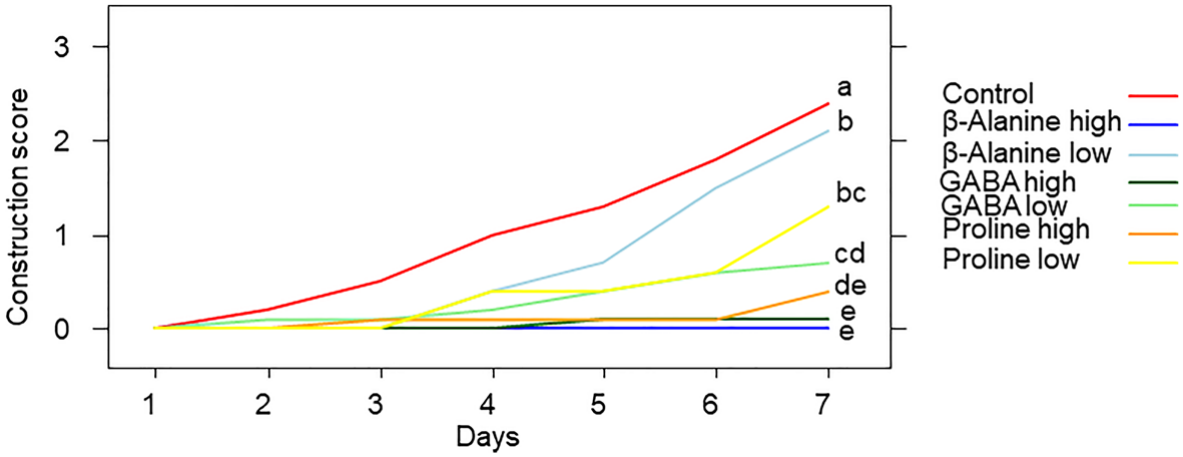
Effect of the amino acids at two different concentrations in the diet (low: 1 mM, and high: 1 M) on the nest construction process. Different letters indicate a pairwise-comparison with P < 0.05.

The amino acid consumption, their concentrations, and the days since the onset of the experiment interacted to affect the nest construction (Fig. 2, amino acids × concentration × days: F_2,464_ = 7.224, P < 0.001; amino acids × concentration: F_2,464_ = 2.002, P = 0.136; amino acids × days: F_3,464_ = 35.620, P < 0.0001; concentration × days: F_1,464_ = 79.953, P < 0.0001; amino acids: F_3,464_ = 2.449, P = 0.062; concentration: F_1,464_ = 14.526, P < 0.001; days: F_1,9.36_ = 46.216, P < 0.0001). Hornets built their nests faster when fed only with sucrose solution. The higher the concentration of amino acid ingested, the slower the nest construction (Fig. 2). Hornets fed with high GABA and β-alanine concentrations failed to initiate any construction during the experiment.

**Figure 3:**
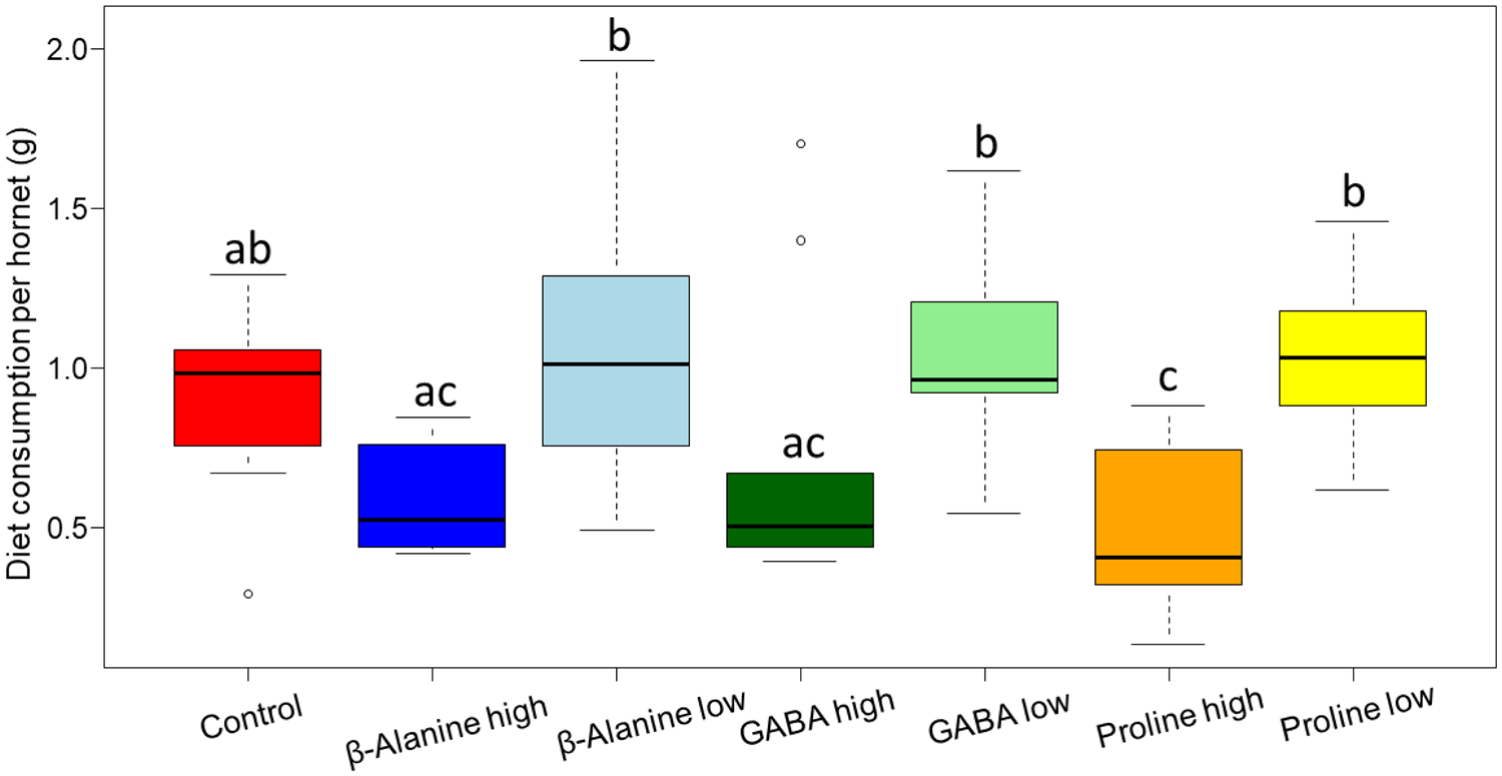
Diet consumption per hornet (g) during 7 days. The boxes represent the first and third quartiles and the median. The whiskers represent the maximum and minimum values. The circles represent the outliers. Different letters indicate a pairwise-comparison with P < 0.05.

**Figure 4:**
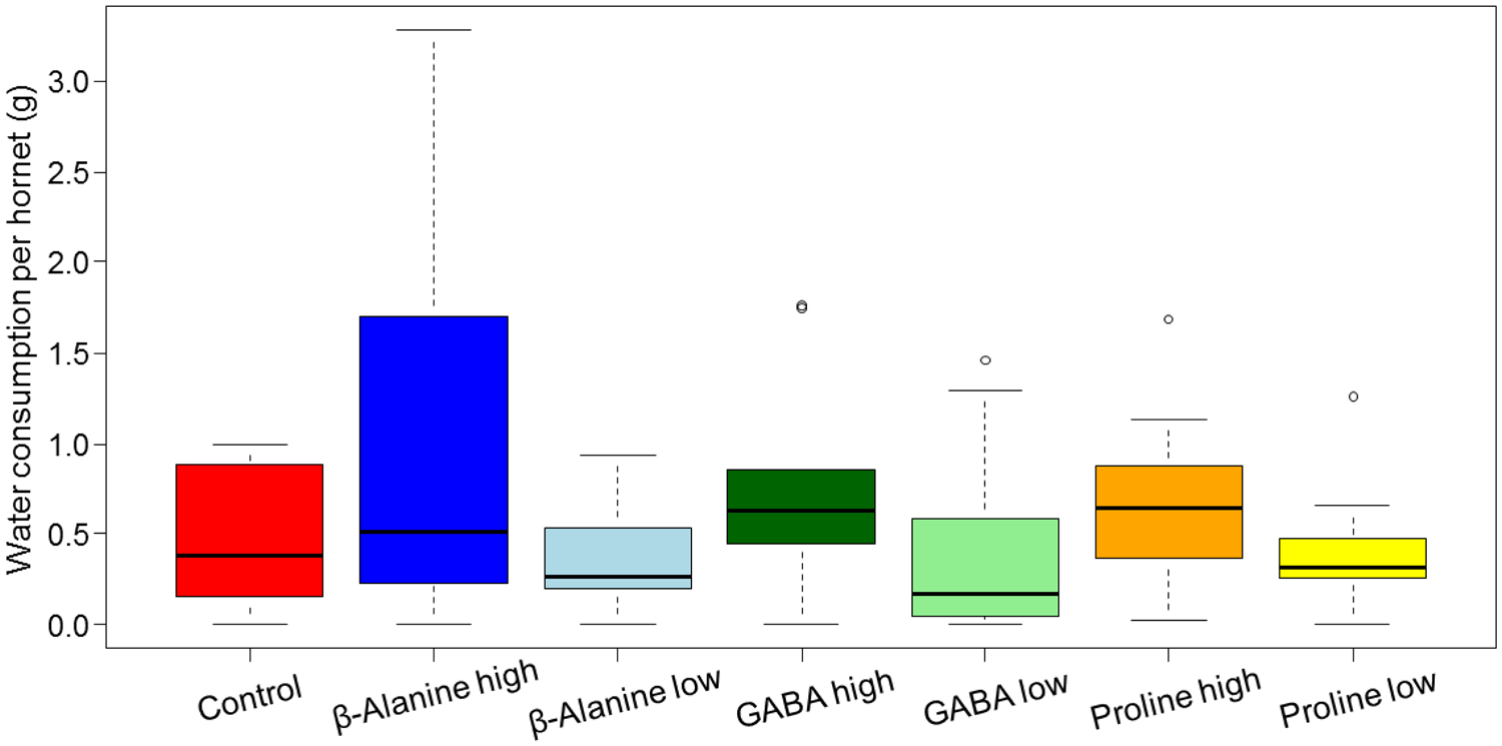
Water consumption per hornet (g) during 7 days. The boxes represent the first and third quartiles and the median. The whiskers represent the maximum and minimum values. The circles represent the outliers. Due to the highly conservative multiple comparison test used, no significant difference among the groups was detected.

Hornets modulated their diet consumption according to the amino acids consumed and their concentration, by drinking less solution when the concentration was high (Fig. 3. Amino acids: F_3,53.8_ = 6.101, P < 0.001; concentration; F_1,52.632_ = 57.063, P < 0.0001; amino acids × concentration: F_2,52.420_ = 0.588, P = 0.559). Hornets also drank more water when they consumed high concentrations of amino acids (Fig. 4, amino acids: F_3,53.7_ = 0.851, P = 0.471; concentration: F_1,52.2_ = 8.602, P < 0.01; amino acids × concentration: F_2,52.1_ = 0.203, P = 0.816).

### Experiment 2: Allocation of amino acids in the hornet body

**Figure 5:**
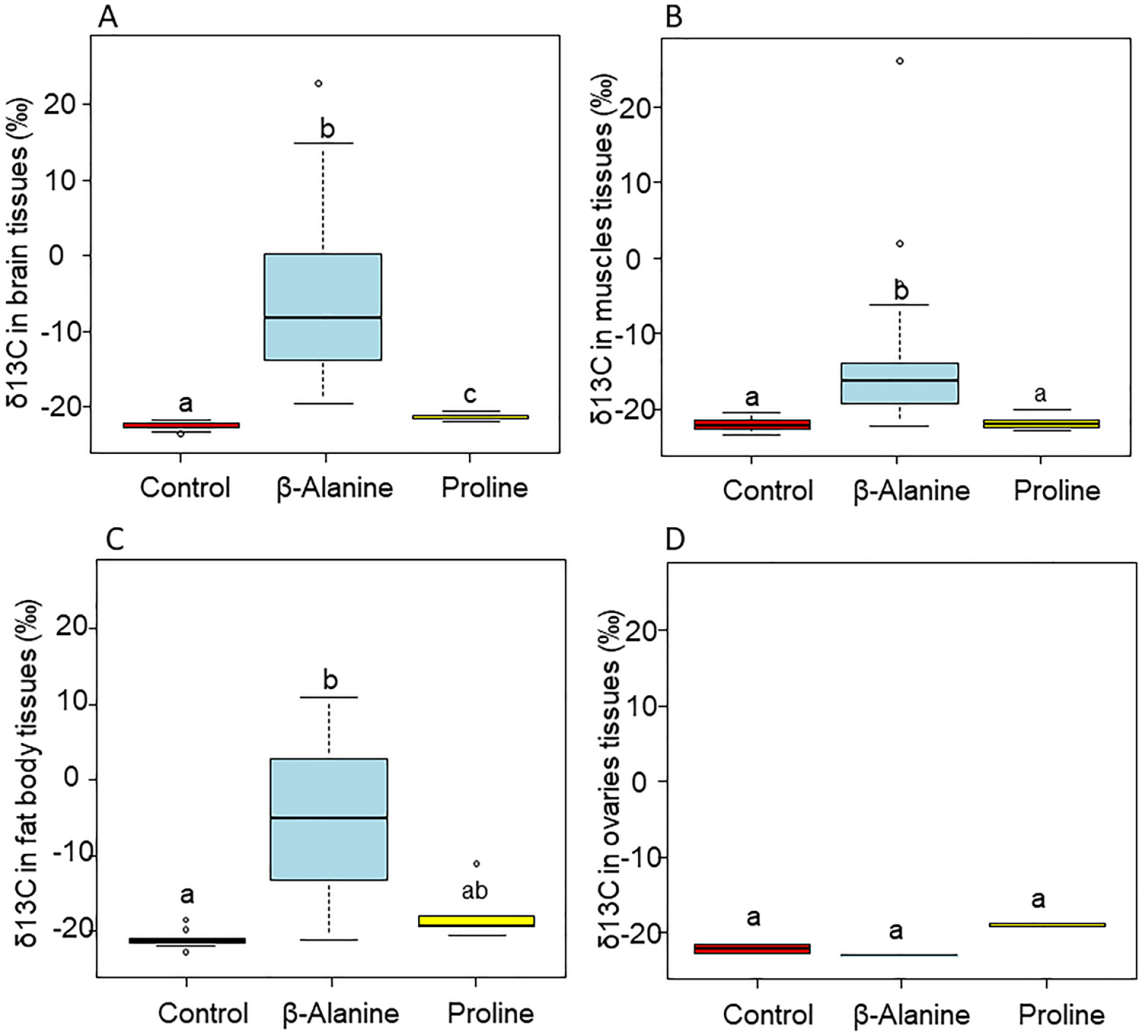
δ^13^C values of hornet body tissues fed with ^13^C-β-alanine and ^13^C-proline, and their control. (**A**) brain tissues, (**B**) muscle tissues, (**C**) fat body tissues, and (**D**) ovarian tissues. Note that lower negative or positive δ^13^C values indicate higher tracer content. The boxes represent the first and third quartiles and the median. The whiskers represent the maximum and minimum values. The circles represent the outliers. Different letters indicate a pairwise-comparison with P < 0.05.

The δ^13^C values differed among the groups for the brain, muscle, and fat body tissues, but not for the ovarian tissues (Fig. 5, brain: F_2,34_ = 22.443, P < 0.0001; Muscles: F_2,81.621_ = 63.476, P < 0.0001; Fat body: F_2,16_ = 7.805, P < 0.01; ovaries: F_2,2_ = 12.309, P = 0.0751). In general, hornets fed with β-alanine had more δ^13^C in their tissues.

## Discussion

We tested the effect of three amino acids common in the natural diet of hornets (proline, GABA, and β-alanine) on hornet survival, nest construction behavior, and food and water consumption. All three amino acids negatively affected the nest construction behavior of the hornets, while high concentrations of dietary β-alanine and GABA also significantly reduced their survival. Using nutrients labeled with carbon stable isotopes, we showed that β-alanine, but not proline, is assimilated and allocated in the different body organs.

Amino acids in nectar are considered to enhance the fecundity and longevity of nectarivore insects^3,4,14,39^. However, in our study, both β-alanine and GABA in the diet reduced hornet lifespan at high concentrations, contrasting the finding of previous studies performed on bees^37,38^. Unlike hornets that feed on larvae saliva, flowers (nectar and pollen) represent the only food resources for most nectarivore insects such as bees. Consequently, bees might have developed more efficient mechanisms to cope with the toxicity of some amino acids found in nectar. The toxicity of GABA was also demonstrated in the oblique-banded leaf-roller larvae (*Choristoneura rosaceana*)^40^. GABA receptors target the insecticides dieldrin and fipronil^41^, and plants use GABA as a first-line defensive mechanism by increasing its content in their tissues in response to insect herbivory. When ingested, the elevated GABA levels in plants become toxic to herbivorous insects^42^. At low concentrations, however, dietary β-alanine and GABA did not affect hornet survival. The toxicity of proteins and essential amino acids at high concentrations has been well documented in social insects^12,43–46^.

High concentrations of dietary β-alanine and GABA inhibited the construction behavior of the hornets, and high proline concentration delayed it. Moreover, the nest construction was slower in hornets fed with low concentrations of these amino acids than in those provided with sucrose solution only; although there was no effect of low concentrations on their survival. Oriental hornets can initiate nest construction by a group of only two individuals^21^. Therefore, the absence or delay of nest construction observed for hornets fed with high concentrations of β-alanine, GABA, and proline cannot be explained by the higher mortality induced by these diets. Our results contrast Ishay’s finding^21^ that hornets could not build when fed with sucrose solution only^21^. A difference between the current study and that by Ishay is our use here of foragers rather than newly-emerged workers. Therefore, if ingestion of a protein source is necessary for nest construction, the consumption of protein or amino acids in the early stages of life appears to be sufficient to initiate it.

GABA reduces neuronal excitability in the nervous system^15^; and β-alanine is expected to have a similar effect, as it activates the GABA receptors^15^. In honeybees, injections of GABA or GABA agonists reduced the level of activity^47^. In vertebrates, activation of GABA receptors induces sleep^48^, and high doses of GABA reduce locomotor activity^49,50^. Similar results were found in rats and birds injected with β-alanine^51,52^. Interestingly, although not considered a neurotransmitter, proline also induces a sedative effect when injected into birds^53^. Thus, the negative impact of proline, β-alanine, and GABA, observed on nest construction in hornets, could be due to decreased locomotor activity.

Proline, β-alanine, and GABA also affected the total food and water consumption of hornets. Individuals consumed less diet and tended to drink more water when these amino acids were present in high concentrations. Animals can regulate their intake of nutrients by over- or under-consumption of particular foods^1^. In our experiments, high concentrations of these amino acids negatively affected both survival and nest construction behavior, it can therefore be assumed that the hornets reduced their food consumption in response to the presence of harmful compounds. The water ingested dilutes the concentration of amino acids in the hemolymph^54^. Thus, by drinking more water, the hornets were likely able to reduce the toxic effects of the amino acids consumed.

We found that β-alanine was allocated in high quantities in most body tissues. Our results confirmed a previous study demonstrating high quantities of β-alanine in the *Drosophila* fly visual system^55^. β-alanine is the precursor of carnosine, found abundantly in skeleton muscles in vertebrates^56^. The high content of β-alanine found in the hornets’ muscles suggests a similar role in insects. Our results also revealed a high content of this amino acid in the fat body tissue. The fat body’s primary role is energy storage, but this tissue also stores excess nutrients and synthesizes proteins and free amino acids, such as proline^57^.

In contrast, we found only a minute quantity of labeled proline in the hornet’s brain. In addition to its role in carbohydrate metabolism^31^, proline can also be easily degraded to glutamate^58^, another important neurotransmitter in insects^15^. Thus, our results indicate that proline is not stored in body tissues and is probably rapidly metabolized.

The findings from this study reveal the toxic effects of the dietary amino acids proline, β-alanine, and GABA on the survival and nest construction behavior in hornets. These amino acids are particularly harmful at high concentrations and, in response to these adverse effects, the hornets consumed less of the diet and drank more water. The fact that proline is not stored in body tissues and is probably rapidly metabolized could explain why proline is less harmful than β-alanine and GABA, even at high concentrations.

In the hornet larval saliva, the maximum concentrations of proline, β-alanine, and GABA, were 14.63 mM, 0.63 mM, and 0.26 mM, respectively^19^. These concentrations are lower than the maximum concentrations used in our experiment and those found in nectar^6,7^, and, therefore, should not reduce an individual’s lifespan. Thus, despite the negative effect of these amino acids at low concentrations on their nest construction behavior, their adaptive roles cannot be rejected. Positive effects at low concentration might impact other behavioral and/or physiological traits. For instance, when fed with a solution extracted from larval saliva, hornets were less aggressive than those provided with sugar solution only^19^. When used as dietary supplements in humans, proline enhances the immune system and boosts wound healing^59^; GABA is used to regulate sleep and prevent depression^60^; and β-Alanine increases physical performances^61,62^. However, the mechanisms of action of these supplements are not always clear^60^, and investigating their effects in simpler organisms such as insects could enable a better understanding of their actions. Additional studies examining the effects of these amino acids on aggressiveness, sociality, and foraging behaviors on hornets should further clarify their ecological roles.

## Material and methods

### Experiment 1: Hornets construction behavior, survival, and nutrient and water consumption

Four colonies of Oriental hornets were used in this experiment. The colonies were collected from the area around Tel Aviv University and each was maintained in a wooden box (14 cubic liters) with a front glass wall, in climate-controlled rooms (25 ± 2 °C, 75 ± 10% RH) with open access to the outside, allowing the workers to forage freely.

Foragers were collected at the colonies' entrances using a sweeping net during August and September 2020. Individuals were placed in groups of seven in experimental colonies confined in wooden boxes (10 × 14.4 × 12 cm) with two transparent Plexiglas walls to allow observations. In the absence of a queen, a small group of workers (5–12) is able to build a nest in the same manner as in queenright colonies^21^. To facilitate the nest construction and provide building materials, we glued a piece of cardboard to the ceiling of each box, which was also supplied with a test tube containing 50% sucrose solution, an additional tube of water, and building materials (soil and paper).

In each group, the sucrose solution contained one of the three amino acids (proline, β-alanine, or GABA) (Sigma-Aldrich), at either high (1 M), or low (1 mM) concentration. These concentrations were chosen to represent the high and low concentrations that naturally occur in nectar plants^6,7^. Control groups were provided with sucrose solution only. To assess the food and water consumption of the hornets, we weighed the tubes containing the diet solution and water at the beginning and the end of the experiment. Control tubes containing the diet solutions and water were weighed in the same manner and placed in boxes without hornets to assess the evaporation rate. Dead hornets were counted and removed daily, and the nest construction was evaluated according to stages observed as follows: Stage 0 corresponded to no construction; Stage 1 to the initiation of construction when stains derived from the building materials started to appear on the ceiling of the boxes; Stage 2 to the construction of a stem; Stage 3 to the construction of the first cell; Stage 4 to the construction of two cells; Stage 5 to the construction of three cells; and Stage 6 corresponded to the construction of four cells. Ten replicates were carried out per group, yielding a total of 490 hornets.

### Experiment 2: Allocation of amino acids in the hornet’s body

Two colonies were used for this experiment: foragers were collected and placed in experimental colonies as previously described. The sucrose solution was enriched with 1 mM of one of the two ^13^C-labeled amino acids, proline or β-alanine (Cambridge Isotope Laboratories, Tewksbury, MA, USA). Control groups were provided with sucrose solution only. Two replicates were carried out per group.

After 7 days, the hornets were killed by freezing, and the different tissues (brain, muscles, fat body, and ovaries) were dissected, collected, and dried at 60 °C for 3 days. Samples of 1 mg of each dry tissue were loaded into tin capsules. For the muscle samples, three replicates were used for each individual. For ovaries and fat body tissues, because the sample masses were insufficient for individual analysis, we pooled the tissue of several individuals (from one to seven) to reach 1 mg of dry mass. The δ^13^C (‰) values in the samples were assessed using a Picarro G2121-i Cavity Ring-Down Spectroscopy δ^13^C stable isotope analyzer with an A0502 ambient CO_2_ interface, an A0201 Combustion Module, and an A0301 gas interface (CM-CRDS)^63^. All ^13^C concentrations are expressed in δ^13^ C_VPDB_.

### Statistical analysis

Survival analysis was performed using a Cox proportional hazards regression model considering censured data. The model included the nutrient type, the concentration, and their interaction, with the colony as a random factor.

For the construction behavior, a score was attributed for each stage of construction (Stage 0 = 0, Stage 1 = 1, Stage 2 = 2, etc.). We used a linear mixed-effects model to compare the evolution of construction between groups followed by a post-hoc pairwise comparison (Tukey adjusted). The model was fitted by specifying the fixed effects (type of nutrient: proline, β-alanine, GABA, or sucrose only; concentration: 0 M, 1 mM, or 1 M; days; and their interactions); the replicates were nested in the colony as a random factor, and days were accounted for repeated measurements. The construction score was transformed (log10(x + 1)) to fit a Gaussian distribution.

The diet and water consumptions were analyzed using a linear mixed-effects model followed by a post-hoc pairwise comparison (Tukey adjusted). The model included the nutrient type, the concentration, and their interaction. The replicates were nested in the colony as random factors. Log_10_ transformations were used for these analyses owing to deviation from a normal distribution.

To study the β-alanine and proline allocation in the hornet body, we used linear mixed-effects models followed by post-hoc pairwise comparisons (Tukey adjusted) for each tissue analyzed (brain, muscles, fat body, and ovaries) to compare the δ^13^C concentrations between groups. The replicates were nested in the colony as a random factor. We used a log_10_ (x-minimum value) transformation to analyze the δ^13^C concentrations owing to deviation from a normal distribution.

For all models, we used the function *lm*er from the R package *lme4*^64^. Statistical tests were run, and graphics were generated on R 4.0.3^65^. Statistical p-value was considered significant under 0.05.

## Data Availability

The data that support the findings of this study are openly available in Zenodo at 10.5281/zenodo.5810231.
